# Electrophysiological Study of Supraspinal Input and Spinal Output of Cat's Subnucleus Reticularis Dorsalis (SRD) Neurons

**DOI:** 10.1371/journal.pone.0060686

**Published:** 2013-03-27

**Authors:** Patricia Velo, Roberto Leiras, Antonio Canedo

**Affiliations:** Department of Physiology, Faculty of Medicine, Santiago de Compostela, Spain; Hospital Nacional de Parapléjicos, Spain

## Abstract

This work addressed the study of subnucleus reticularis dorsalis (SRD) neurons in relation to their supraspinal input and the spinal terminating sites of their descending axons. SRD extracellular unitary recordings from anesthetized cats aimed to specifically test, 1) the rostrocaudal segmental level reached by axons of spinally projecting (SPr) neurons collateralizing or not to or through the ipsilateral nucleus reticularis gigantocellularis (NRGc), 2) whether SPr fibers bifurcate to the thalamus, and 3) the effects exerted on SRD cells by electrically stimulating the locus coeruleus, the periaqueductal grey, the nucleus raphe magnus, and the mesencephalic locomotor region. From a total of 191 SPr fibers tested to cervical 2 (Ce2), thoracic 5 (Th5) and lumbar5 (Lu5) stimulation, 81 ended between Ce2 and Th5 with 39 of them branching to or through the NRGc; 21/49 terminating between Th5 and Lu5 collateralized to or through the same nucleus, as did 34/61 reaching Lu5. The mean antidromic conduction velocity of SPr fibers slowed in the more proximal segments and increased with terminating distance along the cord. None of the 110 axons tested sent collaterals to the thalamus; instead thalamic stimulation induced long-latency polysynaptic responses in most cells but also short-latency, presumed monosynaptic, in 7.9% of the tested neurons (18/227). Antidromic and orthodromic spikes were elicited from the locus coeruleus and nucleus raphe magnus, but exclusively orthodromic responses were observed following stimulation of the periaqueductal gray or mesencephalic locomotor region. The results suggest that information from pain-and-motor-related supraspinal structures converge on SRD cells that through SPr axons having conduction velocities tuned to their length may affect rostral and caudal spinal cord neurons at fixed delays, both directly and in parallel through different descending systems. The SRD will thus play a dual functional role by simultaneously regulating dorsal horn ascending noxious information and pain-related motor responses.

## Introduction

The subnucleus reticularis dorsalis (SRD), also known as the dorsal reticular nucleus, is constituted exclusively by nociceptive neurons [1], [2] reciprocally connected with the noxious region of the spinal dorsal horn in the rat [3]–[9]. Descending projections from the rat's SRD travel in the dorsolateral funiculus to reach the entire rostrocaudal extent of the spinal cord [10], [11] and SRD projections to structures related to pain and motor modulation have also been described in rodents, including the periaqueductal gray (PAG), the locus coeruleus (LC), the nucleus raphe magnus (NRM), the mesencephalic locomotor region (MLR), the nucleus reticularis gigantocellularis (NRGc), the oral part of the spinal trigeminal nucleus, and the thalamus [10], [12]–[17]. It has been reported that more than half of the rat's SRD cells projecting to the thalamus also projected to the spinal cord, thus hypothetically providing simultaneous noxious influence at both levels [16].

We have recently shown that 40 to 60% of SRD neurons send axons to the cat's cervical spinal cord [18], [19] but the proportions of axons, if any, reaching thoracic and/or lumbar segments have not been reported in felines. Accordingly, the first aim of the present work was to elucidate this issue by electrically stimulating the spinal ipsilateral dorsolateral funiculus at cervical, thoracic and lumbar levels.

A second aim was to study whether the cats SRD neurons respond antidromically to electrical stimulation of the somatosensory and/or medial thalamus. This is still an unsolved issue as previous studies in felines showed controversial results related to SRD ascending projections, since whereas few and scattered cells were stained in the SRD after injecting horseradish peroxidase into the thalamus [20], [21], it was latter reported that about half of neurons sampled in and around the SRD responded antidromically to stimulation of the thalamic nucleus centralis lateralis [22].

The effects induced by electrically stimulating other regions known to receive SRD projections in the rat (LC, PAG, NRM, MLR, NRGc) were also studied.

## Materials and Methods

### Ethics Statement

All procedures conformed to the International Council for Laboratory Animal Science, the European Union Council Directive (86/609/EEC), were approved by the University of Santiago de Compostela animal care Committee and were in accordance with the guidelines of the International Association for the Study of Pain [23]. All surgery was performed under anesthesia, and all efforts were made to minimize suffering.

### General

Data were obtained from 28 male cats, weighing 2.7–4.3 kg, under anesthesia and neuromuscular blockade. Surgical anesthesia was induced with ketamine HCl (10–20 mg kg^−1^ I.M.) and continued with α-chloralose (60 mg kg^−1^ I.V.). Additional doses of anesthesia (1/2 of a full dose) were regularly administered every 5–7 h. The depth of anesthesia was evaluated by continuously monitoring the heart rate (maintained around 120 beats min^−1^), the electrocorticogram (ECoG, digitally filtered at a frequency band-pass of 1 to 50–100 Hz) and by observing the state of the pupil. High-amplitude and low-frequency electrocorticographic waves (recorded through an electrode inserted 1–1.5 mm deep in the lateral tip of the cruciate sulcus) were taken as sign of adequate anesthesia, and dilated pupils or pupils reacting rapidly to electrical stimuli were considered to reflect inadequate anesthesia in which case a supplementary half of a full dose of α-chloralose was immediately injected. Tracheal and venous cannulae were inserted; the animal was positioned in a stereotaxic frame and artificially ventilated. After the ECoG showed typical signs of deep general anesthesia, neuromuscular transmission was blocked using vecuronium bromide (0.2–0.3 mg kg ^−1^ h^−1^ I.V.) dissolved in a pH-balanced solution of 5% glucose in physiological saline which was continuously infused (4 ml h^−1^) through a tail vein. A bilateral pneumothorax was routinely performed, the expired CO2 was maintained at 4±0.3%, and the temperature was maintained near 37.5 °C via a DC heating pad under control of a rectal thermoprobe. The foramen magnum was exposed and the posterior arch of the atlas and the occipital bone were resected to uncover the cerebellar vermis. The dura and arachnoid were then removed to insert recording electrodes from the obex to about 3.5 mm caudal to it.

#### Electrical stimulation and extracellular recording

In a first series of experiments (n = 6), bipolar stimulating electrodes, 1 mm inter-tip separation, were placed ipsilaterally in the NRGc (Horsley-Clarke coordinates, AP -7 to -9; L 1 to 2; V 3 to 5 mm from the floor of the fourth ventricle) and, under visual guidance, at the dorsolateral funiculus at cervical (Ce2; mean distance to SRD = 27.6±5.8 mm), thoracic (Th5; mean distance to SRD = 137.5±22.6 mm) and lumbar (Lu5; mean distance to SRD = 293±16.6 mm) spinal cord for antidromic activation of SRD neurons to ascertain the spinal level reached by the SRD descending axons and whether there is some preference for axons collateralizing to or through the NRGc to terminate at a particular spinal level. Antidromicity was determined on the basis of a discrete all-or-none response at threshold stimulating currents (T: intensity evoking a response in ∼50% of stimulus presentations), a constant latency response at 2T stimulating currents, and the ability to follow a 2T train at 150 to 500 Hz of at least three stimuli with constant latencies. Thresholds were abrupt, with less than 0.1 ms change in latency with increasing amplitude for 0.05–0.15 ms duration stimuli. All units fulfilling these criteria also collided with spontaneous or orthodromically-evoked spikes at an interval equal or slightly shorter than the sum of the antidromic latency plus the axonal refractory period and were considered as antidromically activated. In the collision tests, the spontaneous and/or orthodromically-evoked spikes were timed to occur before the expected time of the antidromic spike by an interval greater than the refractory period of the cell. However, for fast conducting axons the intervals at which collision should occur when stimulating through the cervical electrode were short and likely to be close to or to overlap the refractory period. In these cases, Antidromicity mostly relied on the rest of criteria [24]. Furthermore, since the great majority of these fast neurons projected further down the spinal cord, intervals at which orthodromic spikes collided with antidromic responses evoked from Lu5 and/or Th5 were well over the somatic refractory period. Thus, orthodromic collision with Lu5-and/or-Th5-evoked antidromic spikes at intervals longer than the soma refractory period as well as reciprocal collisions between the different spinal stimulating sites, including Ce2, indicated that a single and the same axon was antidromically activated at Ce2, Th5 and/or Lu5 levels.

We assumed that antidromic spike initiation followed the terminating edge of the stimulus pulse [25], [26], [27] and thus antidromic latencies were determined by subtracting pulse duration from the time interval between the beginning of the stimulus artifact and the onset of the antidromic action potential (latency measurements were scored to the nearest 0.01 ms and rounded to the nearest 0.1 ms). Although it is generally assumed that utilization time might vary between 0.1 ms and 0.2 ms, those of reticulospinal neurons have been shown to range between 0 ms and 0.4 ms [28] and are negligibly small for optic tract stimulation [29].

Antidromic activation at Ce2, Th5 and Lu5 levels of the cord served to determine the antidromic conduction velocity for different portions of single axons reaching thoracic and lumbar segments. Measurements of antidromic latencies and of conduction distances allowed calculation of antidromic conduction velocities between the stimulating sites. Mean antidromic velocities were calculated using single-point and two-point stimulation estimates and are expressed as arithmetic means ± SD (Standard Deviation). The statistical comparisons (significant *P* values ≤0.05) derived from the non-parametric Kruskal-Wallis test. The antidromic conduction velocities estimated by stimulating along the descending pathway (Ce2, Th5, Lu5) were combined into different groups to test whether medians between groups were different, under the assumption that the shapes of the underlying distributions were the same. A maximum of six groups per test were used to decrease the chance of a family wise type 1 error (false positive) at the cost of increasing the chance of a type 2 error (false negative). The pairwise comparisons were then conducted using the Dunn's test.

In a second series of experiments (n = 10), stimulating electrodes were placed visually in the cervical dorsal horn at Ce2–Ce3 at a mean distance of 28±5.3 mm to the ipsilateral recording in SRD site, and stereotaxically [25] in the NRGc and the mesencephalic locomotor region (MLR: AP2, L4, V-1 to -2) ipsilaterally to the recording in the SRD; and contralaterally in the medial lemniscus (ML: AP2, L4.5, V-5) and the thalamic nuclei: centralis lateralis (CL: AP9, L4, V4), ventralis medialis (VM: AP9, L2, V1), ventralis posterolateralis (VPL: AP9, L7, V1), and ventralis posteromedialis (VPM: AP9, L5, V1).

Finally, in a separate series of 12 experiments, stimulating bipolar electrodes were placed, ipsilaterally to the SRD recording site, in the locus coeruleus (LC: AP-2, L2 to 2.5, V-1.5 to -1.7), the periaqueductal grey matter(PAG; dorsomedial: AP3.5, L0.2, V2.5; anterior dorsolateral: AP4, L1, V1.5; middle dorsolateral: AP2, L1.5, V1.5; posterior dorsolateral: AP0, L1.8, V1.2; and ventral: AP2, L0.9, V0.3), the nucleus raphe magnus (NRM: AP-6 to -7, L0. V-8 to -9), and the NRGc. No spinal cord stimulation was applied in this series.

Different combinations of stimulating electrodes were used in individual animals of the last two experimental series. Rectangular pulses of 0.01–0.15 ms (typically 0.05 ms) duration and up to a maximum of 2 mA were applied to all the stimulating sites. Short duration pulses of 0.01 to 0.05 ms have been widely used to stimulate central fibers [30]–[32] and present some advantages relative to longer duration pulses: 1) short and strong activating currents decrease utilization time while maintaining a constant threshold [33]; 2) large pulse widths would increase the risk of tissue damage due to their high electrical charge density (the present experiments lasted for 48–96 hours); 3) myelinated fibers could respond better to stimulation using short-pulse duration [34] unlikely to activate cellular somas with larger membrane resistances and longer time constants than the nodes of Ranvier (presumably excited by the extracellularly applied electrical shocks).Therefore, short pulses can theoretically present some specificity to activate myelinated axons [35] not affected by the large capacitance of cell bodies; 4) short duration pulses reduce the probability to induce anodal block and/or anodal break excitation [36], [37]; 5) short duration pulses produce smaller shock artifacts enabling observation of short-latency responses; and 6) the estimates of refractory periods are smaller when short duration pulses are used [38].

Bipolar stimulating needle electrodes with inter-tip separation of 5 to 8 mm were also routinely thrust into all four central foot pads to stimulate Aδ and/or C receptors and fibers driving SRD neurons [18], [19], by passing rectangular current pulses of 0.5–1 ms duration and up to 6 mA current intensity.

Extracellular single-unit recordings of SRD neurons, using epoxy-insulated tungsten microelectrodes of 12 MΩ resistance (A-M Systems, Carlsborg, WA, USA), were obtained from the region located ventrally to the cuneate nucleus and extending rostrocaudally from the obex to about 3.5 mm caudal to it, laterally from 1 to 3 mm, and dorsoventrally from 2.5 to 4.5 mm below the dorsal surface [18], [19]. After the electrode passed through the middle main cuneate nucleus, where the neurons had easily identifiable receptive fields responding to light touch, to brushing the skin [18], [19], [39] or to passive muscular manipulation [40], a neuronal silence was always present before entering the SRD. At this stage, the tissue was covered with warm agar and, when solidified, the electrode was further advanced and the search for SRD cells began. The unitary activity was amplified, digitized at 20 kHz through an analog to digital interface (CED 1401 Plus, Cambridge, UK) and stored on computer for further analysis. CED spike2 v.7 software was used to process and analyze the neuronal activity offline.

#### Histology

Following completion of the experiment, positive currents (20 µA for 20 s) were passed through the stimulating and recording electrodes to mark their tip positions by electrolytic lesions. Animals were killed by perfusion fixation with 4% paraformaldehyde, the neural tissues of interest were removed and postfixed. Transverse 50 µm frozen sections were cut, serially mounted, stained with cresyl violet or neutral red, and the locations of recording and stimulating sites determined.

## Results

As earlier reported [18], the cats SRD cells responding to peripheral stimuli (n = 736) had wide noxious peripheral receptive fields being driven by pinching and/or squeezing the skin and/or by intracutaneous 3.5–6 mA electrical stimulating currents applied to the plantar fore-and/or-hind pads. None of the neurons was activated by the non-nociceptive stimuli tested: innocuous cutaneous (air puffs and gentle touching and brushing the skin) or proprioceptive (passive joint movement and muscular palpation) stimuli. A total of 110 non-responsive cells were also encountered intermingled with the responsive ones all over the sampled region, presumably representing a distinct neuronal population receiving thermonociceptive [2], noxious deep and/or visceral input [9]. Although none of the sampled neurons showed activity related to the electrocardiogram (histograms triggered by the R wave) or to the respiratory cycle (capnogram CO2 wave and respiratory pump), the possibility remains that some of these non-responsive neurons were picked up from the solitary complex or the ambiguous/retroambiguous nuclei and, therefore, were excluded from further analysis.

The data are grouped into three distinct subsets being separately described; first we'll describe the segmental termination of spinally projecting (SPr) SRD neurons; second, electrical stimulation at the dorsolateral funiculus in the cervical spinal cord permitted us to ascertain whether SPr cells collateralized to supraspinal structures; and third, the effects induced upon SRD neurons by electrically stimulating supraspinal sites known to be related with pain and motor processing will be reported.

### 1) Rostrocaudal termination of spinally projecting SRD axons

The spinal rostrocaudal extent of single SRD axons was defined by antidromic activation while recording from their cell bodies. Collateralization to the NRGc was ascertained by collision of antidromic spikes elicited by stimulating the axon at the spinal cord and the NRGc collateral.

**Figure 1:**
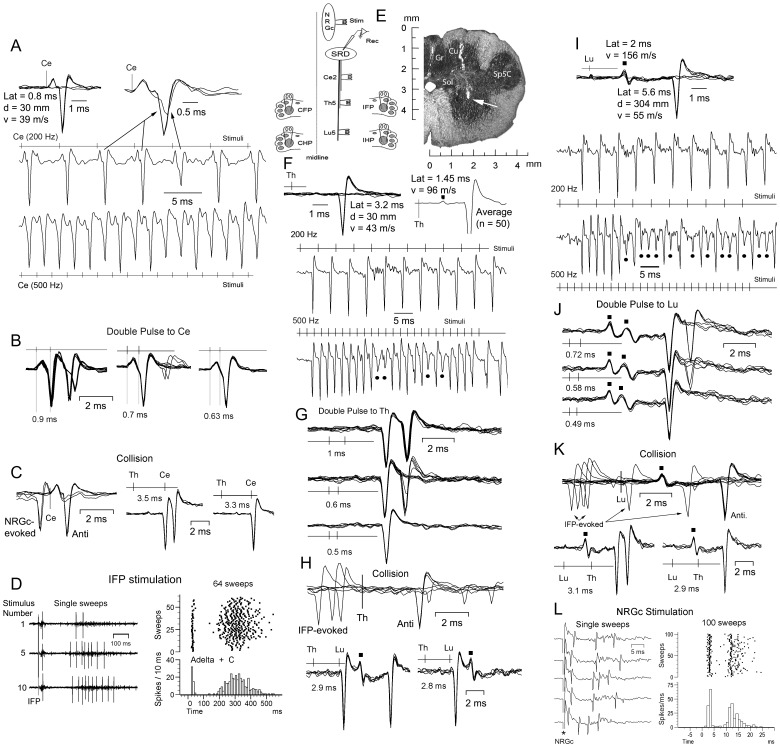
Criteria for antidromic identification. Same silent cell at rest throughout. A, all-or-none responses at threshold current for Ce2 stimulation (upper left) and frequency–following at 200 Hz (middle) and 500 Hz (lower) at whose frequencies some of the spikes showed initial segment (IS)-somatodentritic (SD) breaks (i.e. upper right superimpositions). B, double pulses to Ce2 showed IS-SD breaks at 0.9 ms interpulse interval in part of the superimposed sweeps, with the second pulse being absolutely ineffective when applied within the transmission absolute refractory period. C, collision between NRGc-evoked and antidromic spikes (left) and between Th5-and-Ce2-antidromic spikes (two right panels). D, stimulation of the ipsilateral plantar forepad (IFP) at 1 Hz induced a bimodal response produced by activation of Aδ and C receptors/fibers with the second component (C response) showing windup. E, drawing showing arrangements of stimulating and recording electrodes in this experimental series (left) and a frontal section showing a tract through the nucleus cuneatus (Cu) and the SRD, with the approximate site where this cell was recorded signaled by a white arrow. F, all-none response to Th5 stimulation (upper left) and an earlier response evidenced after averaging 50 responses (upper right, black square). The neuron faithfully followed Th5 stimulation at 200 Hz (middle) and 500 Hz (lower) with the later frequency producing IS-SD breaks or exclusively IS responses (black circles) as stimulation proceeded. G, double pulse Th5 stimulation revealed that at an interpulse interval of 0.5 ms, the second pulse was ineffective. H, collision between IFP-induced orthodromic (upper) and Lu5-evoked antidromic (lower) spikes (the black squares signal the shorter-latency Lu5 response). I, gradual increase in Lu5 stimulation generated a short-latency field potential (notice its increase in amplitude with stimulating current, black square) and a later all-none antidromic response (upper). Both responses followed 200 Hz (middle) and 500 Hz (lower) repetitive stimulation (notice that after the first 6 stimuli at 500 Hz, the majority of antidromic responses failed to invade the soma producing mostly IS potentials (black circles). J, double pulse to Lu5 showing that the second pulse was ineffective at an interpulse interval of 0.49 ms. K, collision between IFP-evoked orthodromic (upper), and Th5-evoked antidromic (lower) spikes. L, NRGc stimulation evoked a bimodal orthodromic response. Latency, distance and single-point estimates of conduction velocity for each stimulating site are shown in A, F and I. Duration of stimulating pulses, A–C: 0.03 ms; D: 1 ms; F–H: 0.04 ms; I–K: 0.05 ms; L: 0.1 ms. Cu, nucleus cuneatus; Gr, nucleus gracilis; IFP, ipsilateral forepad; Sol, nucleus tractus solitarii and tractus solitarius; Sp5C, nucleus trigeminalis spinalis pars caudalis.

**Figure 2:**
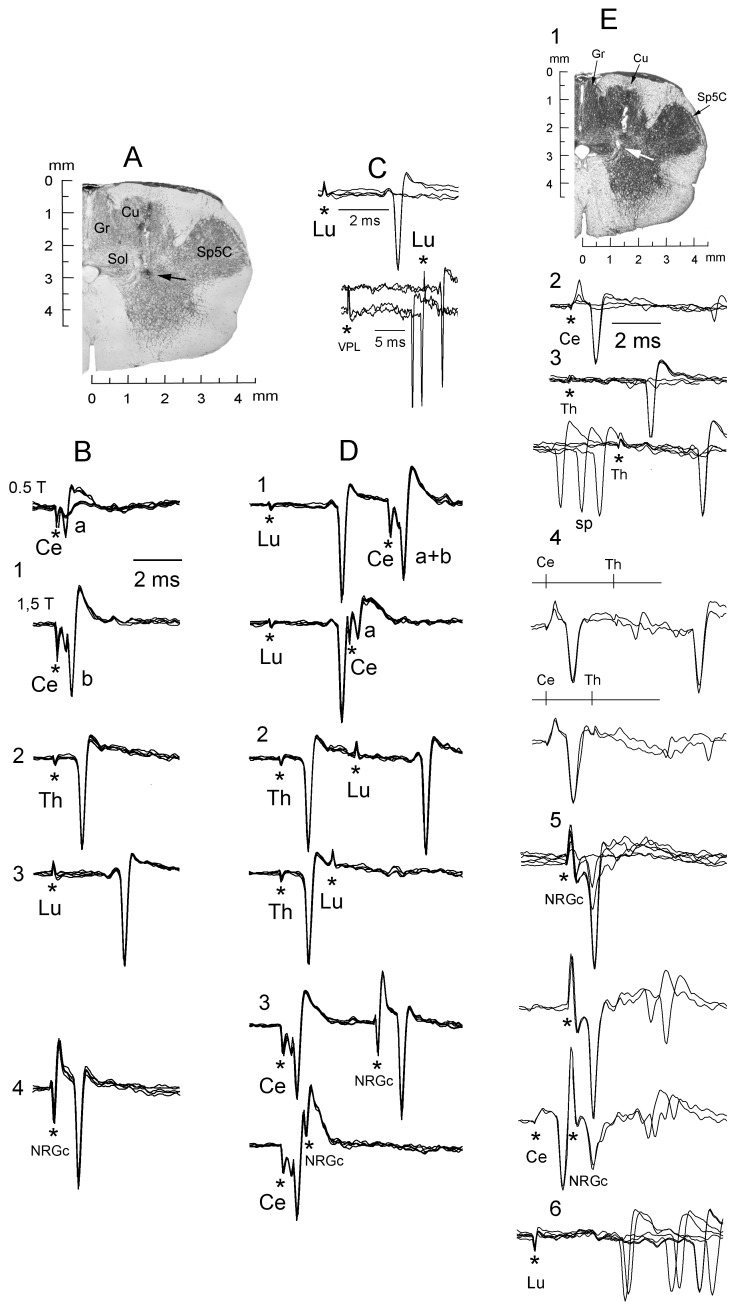
Antidromic identification of spinally-projecting SRD cells collateralizing to or through the NRGc. A, coronal section showing the approximate site (black arrow) where the silent neuron whose responses are illustrated in B–E was recorded. B, superimposed traces showing the fixed antidromic latency of a cell sending an axon up to the lumbar cord and collateralizing to or through the NRGc. Cervical cord stimulation from subthreshold for cell “b” (B1, lower) revealed the presence of a smaller and shorter-latency spike “a” (B1, upper) elicited solely by this stimulating site. C, all-none response to Lu5 stimulation (upper) and collision between IFP-evoked responses and Lu5-antidromic spikes (lower). D, electrical shocks applied along the cord produced antidromic spikes (upper superimpositions in each panel) that collided at the adequate interstimulus interval (lower superimpositions). Note in D1 (lower) that collision of spike “b” uncovered spike “a”. Stimulus artifacts marked by asterisks. E, a different spontaneously active neuron sending an axon up to the thoracic cord recorded at a site signaled by a white arrow (E1). Successive panels with superimposed records show all-none responses to increasing Ce2 stimulation (E2); all-none response to increasing Th5 stimulation (E3, upper) and collision with spontaneous spikes (E3, lower); Ce2-Th5 collision (E4, lower); all-none response to increasing NRGc stimulation (E5, upper) and Ce2-NRGc collision (E5. Lower); and orthodromic responses to Lu5 stimulation (E6). Cu, nucleus cuneatus; Gr, nucleus gracilis; LRt, nucleus reticularis lateralis; IO, nuclei olivaris inferioris; Py, tractus pyramidalis; Sp5C, nucleus trigeminalis spinalis pars caudalis.

The data in Figures 1 and 2 exemplify the criteria followed to antidromically characterize SPr cells not collateralizing (Fig.1) and sending a collateral branch (Fig.2) to or through the NRGc, with the experimental protocol schematized in Fig. 1E (left). The SPr cell recorded at a site signaled by a white arrow in Fig.1E (right) had definite thresholds and followed up to 500 Hz repetitive stimulation at Ce2 (Fig.1A), Th5 (Fig.1F) and Lu5 (Fig.1 I). Thresholds were abrupt, with less than 0.1 msec jitter or change in latency with increasing amplitude for 0.05–0.15 ms duration stimuli. Double pulse stimulation revealed presumed transmission absolute refractory periods of 0.63 ms to Ce2 (Fig. 1B, right) of 0.5 ms to Th5 (Fig. 1G, lower) and of 0.49 ms to Lu5 (Fig. 1J, lower) stimulation. Overall, the axonal refractory period estimates obtained for Ce2, Th5 and Lu5 sites were not significantly different and had mean values of 0.5±0.2 ms for Ce2, of 0.49±0.2 ms for Th5 and of 0.5±0.16 ms for Lu5 (n = 35), suggesting that the excitability of the SPr axons vary little along their descending path. The neuron of Fig.1 was silent at rest as were about 65% (125/191) of the recorded SPr cells, as previously reported [18]. Collision between orthodromically-evoked and antidromic spikes as well as reciprocal collision tests are shown in Figs. 1C, 1H and 1K, as indicated. The neuron had a wide noxious peripheral receptive field generating a bimodal response to 3.5 mA transcutaneous electrical stimulation at all four plantar pads (i.e. Fig.1D for ipsilateral forepad, IFP, stimulation); this response was brought about by Aδ and C nociceptive fiber's activation: there is no Aβ input to the cats SRD [18]. The cell did not respond antidromically to NRGc stimulation but this did induce a bimodal orthodromic response (Fig. 1L). Notice that stimulation at all three spinal sites also generated a smaller-amplitude, shorter-latency response (signaled by black squares). The estimates of antidromic conduction velocity to single-point stimulation were 36 m s^−1^ (Ce2-SRD), 44 m s^−1^ (Th5-SRD) and 55 m s^−1^ (Lu5-SRD); the two-point estimates were 62 m s^−1^ (Th5-Ce2), 77 m s^−1^ (Lu5-Th5) and 68 m s^−1^ (Lu5-Ce2).

The antidromic responses of two neurons (from the same cat) reaching the lumbar cord and collateralizing to or through the NRGc are illustrated in Fig. 2. In the example of Fig. 2B, single-point estimates gave conduction velocities of 74 m s^−1^ (Ce2-SRD), 128 m s^−1^ (Th5-SRD) and 114 m s^−1^ (Lu5-SRD) while two-point estimates gave 182 m s^−1^ (Th5-Ce2), 105 m s^−1^ (Lu5-Th5), and 123 m s^−1^ (Lu5-Ce2) (duration of stimulating pulses: 0.05 ms; distances to the SRD recording site: Ce2-SRD = 37 mm; Th5-SRD = 128 mm; Lu5-SRD = 307 mm). To estimate conduction velocity, antidromic latencies were always measured using 2T currents to minimize utilization time and axonal refractory period. With these stimulus intensities, the Ce2 antidromic stimulus never failed to activate the axons of Th5 cells and the Th5 stimulus never failed to activate the axons of Lu5 cells. Previous work using antidromic stimulation have usually taken utilization times ranging from 0.1 to 0.2 ms [41]–[43]. If 0.1 ms utilization time is taken in the example of Fig. 2B, the single point estimates will increase to 92.5 m s^−1^ for Ce2-SRD, to 142.2 m s^−1^ for Th5-SRD and to 118 m s^−1^ for Lu5-SRD; and if 0.2 ms is used, the velocities will be 123 m s^−1^ (Ce2-SRD), 160 m s^−1^ (Th5-SRD) and 123 m s^−1^ (Lu5-SRD).

Although utilization times were unknown, single point estimates in the whole sample were within the range of reported conduction velocities for reticulospinal cells from the cat's medial pontomedullary reticular formation [44], [45].

An example of a neuron with an axon having conduction velocity within the lower range and that did not reach the lumbar cord is shown in Fig. 2E. Figure 2E1 illustrates the lesion site (white arrow) where this spontaneously active SPr neuron collateralizing to or through the nRGc was recorded. Single-point antidromic velocity estimates were 46 m s^−1^ (Ce2-SRD) and 39 m s^−1^ (Th5-SRD); two-point estimate was 37 m s^−1^ (Th5-Ce2). Antidromic responses and collisions are illustrated through Figs. 2E2-2E5, as indicated. Orthodromic responses induced by Lu5 stimulation are shown in Fig. 2E6.

**Figure 3:**
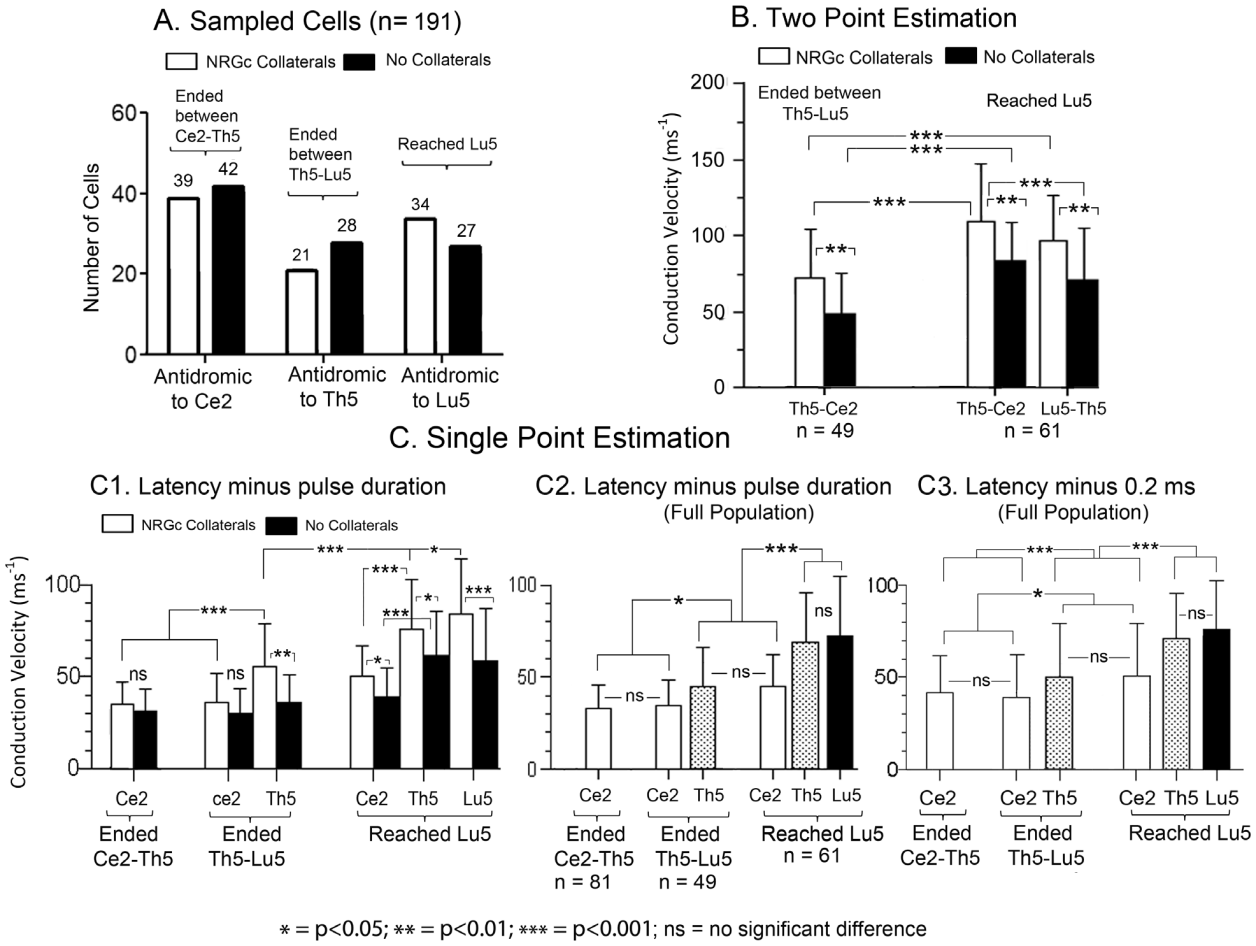
SRD spinally projecting fibers increased in antidromic conduction velocity with termination distance and NRGc collateralization. A, sampled neurons with fibers collateralizing (white columns) or not (black columns) to or through the NRGc, and ending between Ce2-Th5, between Th5-Lu5 or Lu5. B, thoracic-cervical (Th5-Ce2) and lumbar-thoracic (Lu5-Th5) means ± SD antidromic conduction velocities estimated by two-point stimulation. C, means ± SD of antidromic conduction velocities estimated by single point stimulation subtracting pulse duration (C1; C2) or 0.2 ms (C3) to antidromic latency measurements. Abbreviations at the X axis signal the stimulation sites at each spinal segment. The *P*-values of the pairwise comparisons were calculated by the Dunn's post-test.

The full data from this series are graphically summarized in Fig. 3A using the experimental arrangement schematized in Fig. 1E (left). From a total of 191 SPr cells sampled, some 42% sent descending axons terminating rostral to Th5 (antidromic to Ce2 but neither to Th5 nor to Lu5), another 26% of axons ended at some level between Th5 and Lu5 (antidromic to Th5 but not to Lu5), and about 32% reached the Lu5 segment. A total of 39 SPr axons collateralizing to or through the NRGc (39/94: 41.5%) terminated at segmental levels between Ce2 and Th5, representing 48.1% (39/81) of the total amount of axons ending at these levels; 21 reached the Th5 segment (21/94: 22.3%) representing 42.8% (21/49) of SPr axons ending at this level, and 34 reached Lu5 (34/94: 36.2%) which represent 55.7% (34/61) of all axons reaching this segment of the cord.

The mean antidromic conduction velocities increased with axonal termination distance along the cord when estimated by single-point stimulation. However, the mean antidromic velocity of axons reaching the Lu5 segment were faster conducting along the Th5-Ce2 path than between the Lu5-Th5 route when estimated by two-point stimulation (Fig. 3B), the difference being not statistically significant. The two-point estimates of antidromic conduction velocity were non-physiological in a considerable number of cases, ranging from 175 to more than 300 m s^−1^ (n = 26), and thus single-point estimates were considered more accurate. The latency in single point estimates includes a stimulus utilization time and a time delay between the antidromic invasion of the initial segment (IS) and the somato-dendritic (SD) neuronal components. As stated in the methods section, we assumed that the utilization time equalled stimulus duration (Figs. 3C1, 3C2), an assumption that did not suppose a unique utilization time for all sites stimulated but that probably underestimated utilization times, given the short duration pulses used, and thus probably also underestimated conduction velocities. The IS-SD delay is also a critical factor determining antidromic latency. Only very few neurons showed antidromic responses with IS-SD breaks to high-frequency stimulation and/or paired stimuli. Since IS-SD delays are only seen when the extracellular recording electrode is near the axon hillock, it is unknown whether the great majority of recordings (without IS-SD breaks) came from the soma-dendrites, from the axon's initial segment or from both.

The descending fibers collateralizing to or through the NRGc were faster conducting than those that did not issue NRGc collaterals (Figs. 3B and 3C1). When considering that utilization time equalled pulse duration and that the recordings originated from the axonal initial segment (Figs. 3C1, 3C2), the population of SRD descending fibers as a whole had mean conduction velocities to single-point stimulation of 33.4±12.4 m s^−1^ for those terminating above Th5, of 45.15±21 m s^−1^ for fibers terminating between Th5 and Lu5, and of 72.7±31.9 m s^−1^ for the sample reaching the Lu5 segment (Fig. 3C2). If, instead, the assumption is made that utilization time and IS-SD delay taken together, had a value of 0.2 ms, then the population velocities will be 42±20 m s^−1^, 50±29.6 m s^−1^ and 76±27 m s^−1^ for fibers terminating above Th5, between Th5-Lu5 and for those reaching the Lu5 segment, respectively (Fig. 3C3). The data in Fig. 3C show that: 1) conduction velocities along the Ce2-SRD pathway were not significantly different (ns) for collateralizing and no-collateralizing fibers ending above Lu5 (Fig. 3C1). Collateralizing fibers reaching Lu5 were significantly faster than no collateralizing ones; 2) the relative conduction velocities along the descending pathway were similar when estimated by subtracting pulse duration (Fig. 3C2) or 0.2 ms (Fig. 3C3) from the antidromic latencies; 3) the antidromic velocity through the Ce2-SRD route was not significantly different for fibers ending above Lu5, but was significantly faster for fibers reaching Lu5 than for those terminating more rostrally; and 4) the antidromic conduction velocity increased with fiber length: longer fibers were significantly faster than shorter ones. Therefore, the overall outcome of these data suggesting a general conduction slowing along the Ce2-SRD course and increasing mean conduction velocity with termination distance remained unvaried when estimating antidromic velocity by subtracting either pulse duration or 0.2 ms from the antidromic latencies.

The subpopulation of collateralizing fibers to or through the NRGc ending above Th5, between Th5-Lu5, or reaching the Lu5 segment had, respectively, mean antidromic conduction velocities to single-point estimates of 36.8 (46) m s^−1^, of 56 (62) m s^−1^, and of 84 (89) m s^−1^ (values in brackets are the mean velocities after 0.2 ms latency subtraction). The mean velocities of the subpopulation of fibers that did not collateralize to or through the NRGc and ending at the same levels were, respectively, 31 (38) m s^−1^, 37 (49) m s^−1^ and 58 (60) m s^−1^.

### 2) The SRD cells had scarce thalamic projections

**Figure 4:**
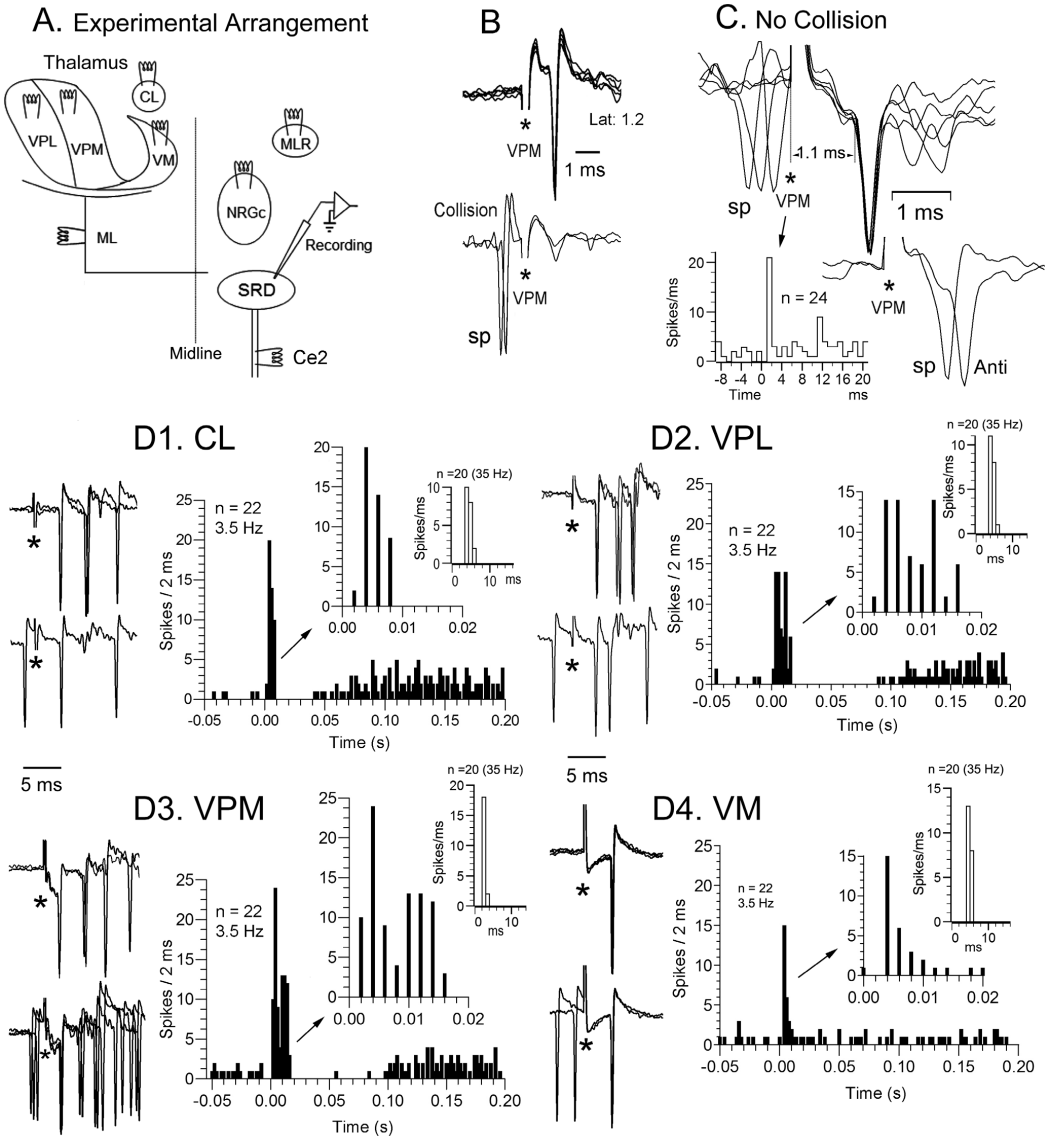
Short-latency thalamic-SRD effects. A, schematic diagram illustrating the experimental design for this series. B, antidromic response to thalamic ventroposteromedial nucleus stimulation (VPM, upper superimpositions) colliding with a spontaneous (sp) spike (lower row). C, VPM stimulation induced a short-latency orthodromic spike (1.1 ms latency) in a different cell which failed to collide with spontaneous spikes (sp) preceding the stimulus by less than 1 ms (upper right) but the VPM-induced orthodromic response was cancelled during the refractory period of a preceding spontaneous discharge (below, right). The peristimulus histogram shown below (VPM stimuli at time 0) was compiled at 3.5 Hz. Stimulus artifacts signaled by asterisks. D, the raw data from another cell illustrate short-latency spikes to stimulation of all 4 thalamic sites tested. Collision with spontaneous spikes did not occur (lower records of each pair of recordings at the left of histograms). The peristimulus histograms at right (stimuli at time 0) were compiled taken the means of spikes to 20 consecutive stimuli at 3.5 Hz (black columns) and 35 Hz (white columns), generated by the three cells presumably responding monosynaptically to all thalamic sites stimulated. The presumed monosynaptic responses at 3.5 Hz appeared at the second 2 ms bin in the black bar histograms, with those spikes at the first bin representing late di-polysynaptic responses induced by preceding stimuli, which disappeared when compiling histograms at 35 Hz (white columns) starting at the fourth stimulus to eliminate responses not following 35 Hz induced by the first one-three stimuli. CL, n. centralis lateralis; ML, lemniscus medialis; MLR, mesencephalic locomotor region; VM, n. ventralis medialis; VPL, n. ventralis posterior lateralis; VPM, n. ventralis posterior medialis.

This series of experiments (experimental arrangement schematized in Fig. 4A) was prompted by previous data from rodents reporting SRD-thalamic projections [15], [17] with more than half of them being collateral branches of descending axons to the spinal cord [16]. The obtained data are resumed in Table 1. Contrary to found in rat, cats SRD axons to the thalamus were uncommon. Two out of 80 SRD cells tested were antidromically fired from the contralateral VPL, and another two neurons out of 60 tested responded antidromically to contralateral VPM stimulation; all four cells sent their axons through the ML and none was antidromically activated from the spinal cord. No antidromic responses were contralaterally elicited from the CL (53 cells tested) or the VM (39 cells tested).

**Table 1:** Cells Tested to Cervical Cord Stimulation.

Stim. Site	Tested	Antidr.	Orth (+)	SPr
MLR	60	0	38	29
ML	101	9*	54	54
NRGc	86	43(25*)	34	54
Thalamus:				
CL	53	0	33	17
VM	39	0	26	16
VPL	78	2(x)	76	57
VPM	57	2(x)	50	20
TOTAL	474	56	311	247

* =  Also antidromically activated from the cervical cord. X =  Also antidromically activated from the Contralateral Medial Lemniscus (ML).

Most SRD neurons were transynaptically activated by thalamic stimulation, probably through the cerebral cortex, although 7.9% of the tested sample (18 out of 227 tested cells: this percentage will increase to 9.7% when considering the 18/185 responsive neurons) showed latencies to the first spike varying from 1.1 to 5 ms [CL (n = 6/53: 11.3%), VM (n = 3/39: 7.7%), VPL (n = 4/78: 5.1%); VPM (n = 5/57: 8.8%)] that appear too short to have been induced through a trans-cortical route (Figs. 4C and D). All these short-latency-responsive cells showed convergence to thalamic stimulation (3 to all thalamic sites stimulated, Fig. 4D); 1 to CL, VPM and VPL; 1 to CL and VPM). The short-latency activation of these convergent cells to CL, VPL and VPM stimulation was followed by a period of decreased or silenced activity lasting 30–80 ms which in turn was trailed by a longer-lasting period of increased activity (Fig. 4D1–D3). On the contrary, VM stimulation induced a short-latency response consisting of single spikes or occasionally doublets (Fig. 4D4). The first spike in the response of these 18 neurons faithfully followed up to 50 Hz iterative thalamic stimulation, having standard deviations differing from the mean across trials (jitter) of less than 0.5 ms at 50 Hz and of less than 0.4 ms to 3.5 Hz when computed to 20 consecutive stimuli, which led us to consider the first-latency responses as monosynaptically produced.

The earlier presumed monosynaptic thalamic-SRD response had a latency of 1.1 ms (Fig. 4C) giving conduction times of 0.65 ms (assuming 0.05 ms utilization time and 0.4 ms synaptic delay) or of 0.6 ms (0.1 ms utilization time, 0.4 ms synaptic delay) giving conduction velocities of 37 m s^−1^ or of 40 m s^−1^ taking a linear thalamus-SRD distance of 24 mm. These velocities are within the range of the thalamocortical fibers [46]. In fact, synaptic delay can be as short as 0.3–0.4 ms, [47]–[49] commonly generated by fibers monosynaptically contacting the soma and proximal dendrites of long-axoned cells [50], [51].

There is, however, the possibility that slower antidromic thalamic-SRD responses could be masked by inhibition following the faster monosynaptic spikes. This is unlikely since such a strong hypothetic inhibition, able to suppress antidromic spikes, should also abolish spontaneous and orthodromically-induced action potentials and will be apparent in peristimulus time histograms such as the one shown in Fig. 4C, where there is no evidence of such an inhibition (see also the expanded histograms in the insets of Figs. 4D1–D4).

None of the 70 SRD cells tested to MLR stimulation responded antidromically and the first latency of the induced orthodromic activity (38 out of 60 tested cells: 63%) averaged 18 ms (range, 12–28.5 ms). Antidromic responses to NRGc stimulation were observed in 50% of the tested SRD cells (43/86) with more than half (25/43) also sending a descending axon to the cord. Finally, 9 out of 101 cells tested were antidromically fired from the contralateral ML (8.9%), five of which were also antidromically fired from the ipsilateral cervical dorsolateral funiculus but not from the thalamus and hence they might have projected either to non-stimulated thalamic sites or to extrathalamic regions.

According to these data, the number of ascending axons coursing through the ML to the thalamus was considerably fewer than previous findings reported in rodents [15], [16] although probably not all the SRD-thalamic fibers course in the ML, particularly those directed to the medial/intralaminar nuclei.

### 3) Effects induced by stimulation of supraspinal pain-and-motor-related structures

Spinal cord stimulation was not applied in this experimental series. The results are summarized in Table 2. Antidromic responses were observed following stimulation of the LC (5.4%), NRM (23.3%) and NRGc (53.2%) but not in response to the PAG sites tested through its rostrocaudal and dorsoventral extent (Table 2).

**Table 2:** Cells Not tested to Spinal Stimulation.

Stim. Site	Tested	Responded	Antidromic	Orth
LC	152	129 (84.9%)	7 (5.4%)	122
dlPAG (ant.)	94	39 (41.5%)	0	39
dlPAG (mid.)	82	35 (42.7%)	0	35
dlPAG (post.)	149	76 (51%)	0	76
dmPAG (post.)	10	6 (60%)	0	6
vlPAG (post.)	42	14(33.3%)	0	14
NRM	41	30 (73.2%)	7 (23.3%)	23
NRGc	138	109 (79%)	58 (53.2%)	51
TOTAL	708	438 (61.9%)	72 (16.4%)	366

**Figure 5:**
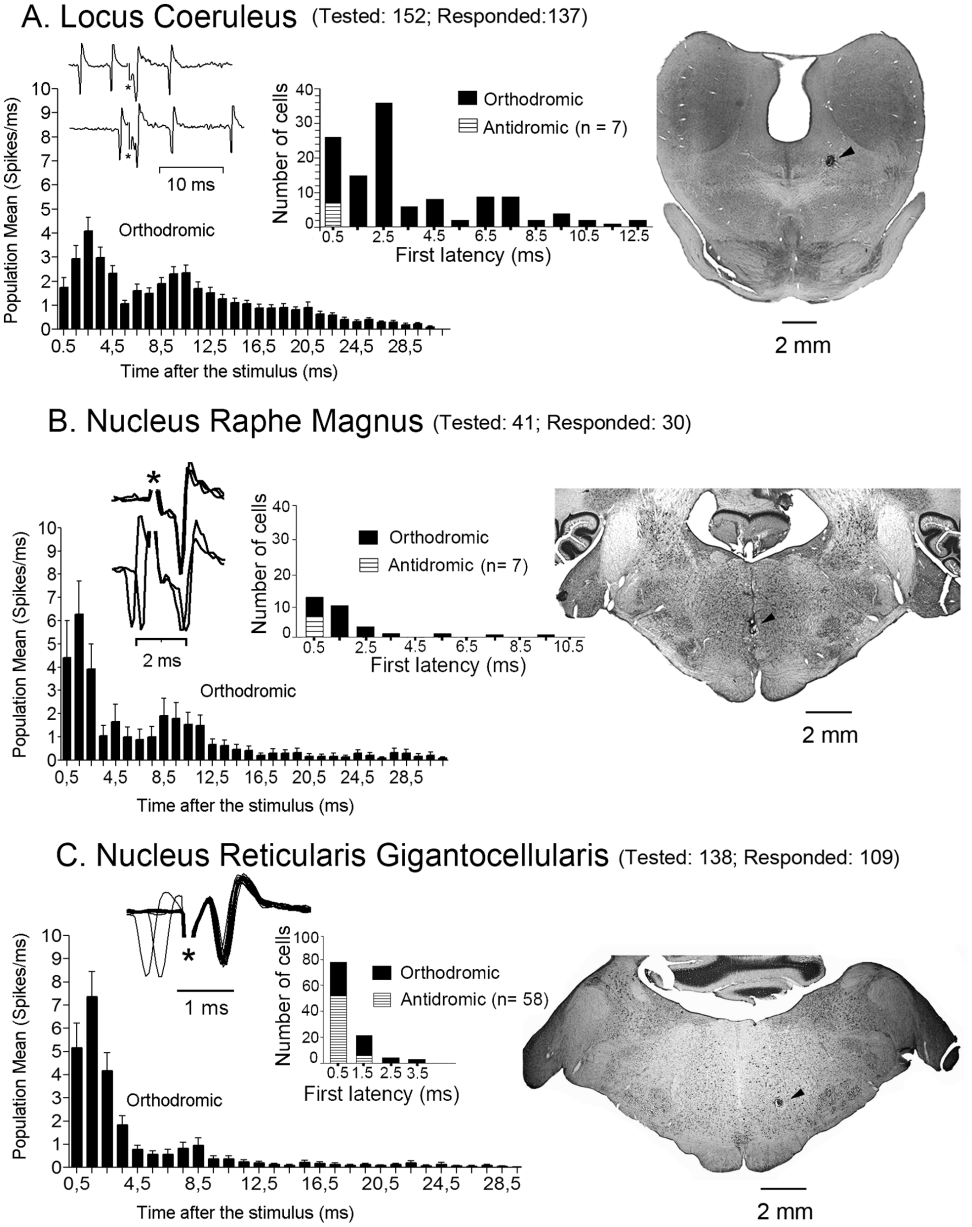
Population responses of SRD cells to LC, NRM and NRGc stimulation. The poststimulus histograms at left were computed with the population means of spikes ± SEM from all cells responding orthodromically. Samples of short-latency responses (lack of collision) are shown over each poststimulus histogram. The latency distribution of the sampled cells are shown in the center histograms of each panel, and representative electrolytic lesions (signaled by arrowheads) made through the stimulating electrodes are shown in the coronal sections at right.

**Figure 6:**
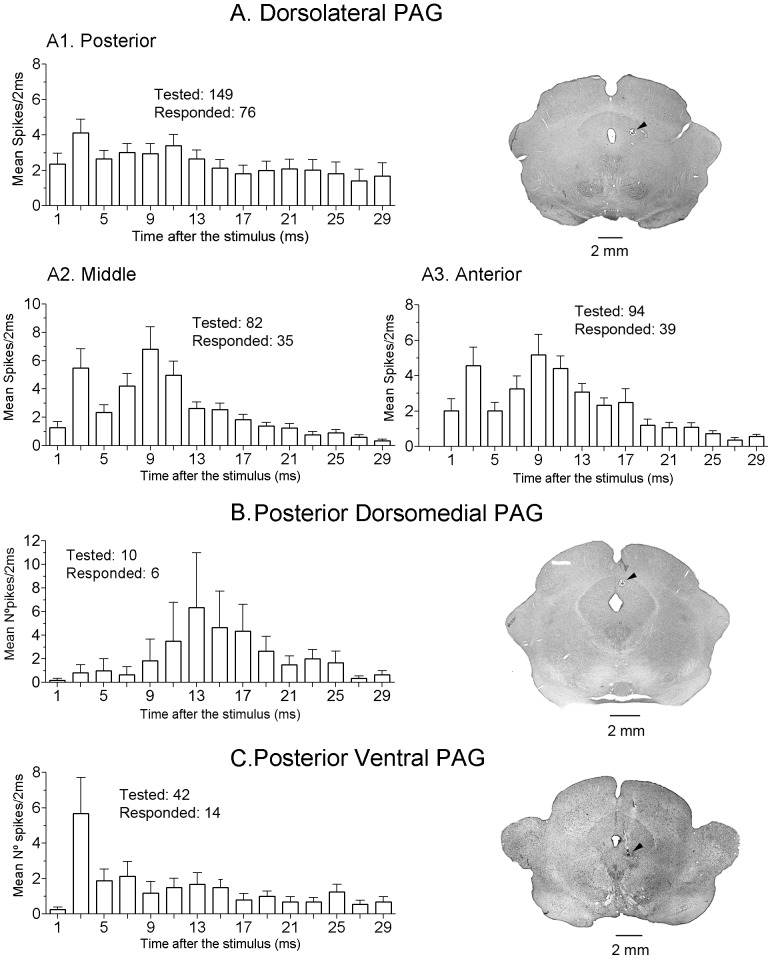
Population responses to PAG stimulation. No antidromic responses were observed. Histograms computed as in Fig. 5. The coronal sections at right show examples of electrolytic lesions (signaled by arrowheads) made at PAG stimulating sites.

The population means of all spikes of the orthodromic responses to 20 consecutive stimuli at a frequency of 3.5 Hz (to avoid wind-up generation) for each stimulating site were grouped in bins of 1 or 2 ms and plotted in Figs. 5 and 6. The resultant response patterns, for the first 30 ms following the stimuli, differed according to the stimulating sites. LC (Fig. 5A) as well as middle and anterior PAG (Fig. 6 A2 and A3) stimulation induced bimodal changes with increased activity peaking at latencies of 2–4 ms and of 9–11 ms followed by a steadily decrease in the induced activity. This bimodal distribution might represent temporal integration of information through different mechanisms such as two different levels of afferent drive, distinct grades of intrinsic excitability [19], presence of inhibitory interneurons shaping the interval between the excitatory peaks, or to a combination of these.

The bimodal behavior was less evident to NRM stimulation which, like NRGc stimulation, induced mostly short-latency responses grouped at latencies up to 3 ms (Fig. 5B and C) due to silenced firing, during 25–60 ms in most cells, following the first-latency responses. The dorsolateral PAG stimulation generated sustained rather than phasic population responses (Fig. 6A), the posterior dorsomedial PAG stimulation induced longer latency activity in the small sample of 6 responsive neurons (Fig. 6B), and the postero-ventral PAG stimulation tended to elicit a unique response at 3–4 ms latency (Fig. 6C). Overall, PAG stimulating sites orthodromically drove 170/377 (45.1%) of SRD cells. Convergence of effects was observed following stimulation of LC and the posterior dlPAG (72 out of 103 tested cells: 69.9%; 3 antidromic to LC); after stimulating LC and NRGc (13 out of 19 cells tested: 68.4%), and following stimulation of NRGc and NRM (24 out of 32 tested cells: 75%; 2 antidromic to both, 9 antidromic to NRGc). Convergence of effects from PAG and NRGc was not tested. Short-latency orthodromic activation (first latency less than 5 ms) following at least 50 Hz iterative stimulation, thus being probably monosynaptic, was observed in response to LC stimulation (42 of 122 responding cells: 34.4%), in response to PAG stimulation (14 out of 170 responding cells: 8.2%), in response to NRGc stimulation (37 of 51 responsive neurons: 72.5%), and in response to NRM stimulation (16 of 23 responding neurons: 69.6%).

## Discussion

The following conclusions can be drawn from the present electrophysiological study: 1) the cats SPr fibers coursing to the spinal cord as far as Lu5 level increased in conduction speed with termination distance and NRGc collateralization 2) very few SRD neurons sent axons to the thalamus, with no SPr fibers collateralizing to the MLR, the PAG or the thalamus, 3) a considerable percentage of SRD cells responding to thalamic stimulation (18/185: 9.7%) showed presumed monosynaptic convergent responses to the thalamic stimulated sites, and 4) SRD cells were antidromically fired from the LC, NRM and NRGc but not from the MLR or the PAG.

### 1) SPr axons

Descending SRD fibers terminated all along the spinal cord as in the rat [16] and, on average, increased in antidromic conduction velocity with termination distance: longer fibers had faster conduction velocity, suggesting that conduction times are tuned to fiber length so that synchronous input to SPr neurons may generate descending activity reaching their spinal terminating sites within a narrow time window. This could be possible if conduction velocity relates to soma size and recruitment order and/or if single SPr axons collateralize along the cord with morphological and/or physiological properties tending to maintain isochronous activity at the different terminals. In fact, various central neural pathways present rather uniform and isochronous axonal delays [52].

The full population of SPr fibers showed a general antidromic velocity decrease through the Ce2-SRD pathway when estimated by subtracting either stimulus duration or 0.2 ms from latency measurements (Figs. 3C2, 3C3). Further, the antidromic conduction velocity through the Ce2-SRD pathway was not significantly different for axons terminating above Lu5, but was significantly faster for axons reaching Lu5, implying that fibers ending at Lu5 or below are faster conducting not only at lower segmental levels but also through the Ce2-SRD route. No previous work addressed SPr cell's conduction velocity but a similar conduction delay within the brainstem was reported for reticulospinal axons from the cats medial pontobulbar region [44], [53] and Peterson et al., [53] suggested that this delay could represent a slower conduction velocity or that the fibers follow a wavier path than at lower levels. Slowing in the axon proximal region has also been reported in motoneuronal axons [54]. For SPr neurons receiving common input, the greater velocity slowing for fibers ending at more rostral levels could tend to synchronize arrival times to rostral-caudal segments.

The results show that both pontobulbar [44], [45], [53] and SRD descending axons share similar ranges in conduction velocity. Considering the whole sample of SPr cells from the first two experimental series, 48.6% of them (119/245) collateralized to or through the NRGc, corroborating previous data also from cats [18], [19]. The NRGc projects to the midbrain and diencephalon [55], [56] and sends descending axons primarily to spinal cord motor nuclei [57], [58] thus participating in motor and reflex functions [59]–[61]. Also, electrical stimulation in and around the NRGc suppresses nociceptive brainstem and spinal reflexes [62], [63] and induces aversive-nocifensive behavioral responses [64], [65].

Since the descending SRD neurons collateralizing to or through the NRGc terminated at all levels of the cord, the outputs of the collateralizing SPr cells would concurrently intervene producing and regulating motor responses (through the NRGc reticulospinal cells terminating in the ventral horn) and modulating dorsal horn ascending nociceptive activity mostly through the SPr cells but probably also through NRGc neurons with descending axons terminating at the spinal dorsal horn [57], [66], [67] if the SRD collaterals influence NRGc-reticulospinal nociceptive cells [68]. This proposed SRD-NRGc link may play a functional role in the coordination of pain-related movements, providing rapid postural adjustments and motor reactions to implement withdrawal and more complex nocifensive (and orienting?) responses that will be later refined by the cerebral cortex through the pyramidal tract [28], [69].

In addition, the SRD descending projections are a critical component of the DNIC (diffuse noxious inhibitory control) pathway [70] by inhibiting multimodal dorsal horn neurons [71], [72]; and because the SRD directly communicates with the LC, NRM and NRGc, parallel descending systems may conjointly modulate spinal ascending nociceptive transmission, DNIC, as well as withdrawal, fighting and fleeing responses by engaging different spinal circuitries [73], [74].

### 2) Neither thalamic nor MLR stimulation produced antidromic responses on SPr neurons

SPr cells did not collateralize to the thalamus in spite that about 8.9% of them responded antidromically to ML stimulation. In antidromic identification, only positive findings can be considered relevant. Failure to demonstrate antidromic invasion may reflect inaccessibility of the axon to the stimulus current because of their location, thickness, stimulus parameters, etc. Furthermore, the rat's SRD-thalamic projection is constituted by thin axons [16] and if the same applies to cats, then antidromic activation could have failed due to the high threshold of the terminal fibers. However, tracing studies found very few brainstem reticulospinal neurons with collaterals to the diencephalon [75], [76], and it was postulated that the information reaching the cat's posterior thalamus from the caudal medulla does not preferentially travel in the ML but is probably relayed through more rostral reticular structures [77]. One such structure could be the NRGc, relaying SRD information not only to medial/intralaminar nuclei but to other thalamic sites as well. In fact, NRGc neurons with ascending axons project to the zona incerta, the medial/intralaminar thalamic nuclei [56], [78], and the dorsal thalamus [58]. Still other NRGc cells have ascending and descending bifurcating fibers [78]–[80], being thus able to spread fast coordinated signals allowing for rapid responses to relevant afferent stimuli.

The thalamus is essentially part of the ascending projection systems, with little descending or reciprocal input to prethalamic regions. Nevertheless, we found that a considerable 7.9% of the tested cells responded at a short and fixed latency to thalamic stimulation which we presume was a monosynaptic effect. Monosynaptic connections have been described from the medial/intralaminar thalamus to the pontobulbar reticular formation [81], [82] and descending projections from the auditory thalamus to the inferior colliculi do exist [83], [84] but, to the better of our knowledge, no other descending thalamic projections have been described. The short-latency (1.1 ms in Fig. 4C) responses, frequency-following and lack of collision (Figs. 4C and D) made us to consider these effects as monosynaptically generated via an unknown pathway. Furthermore, the short-latency effects showed convergence to distinct thalamic stimulating sites (Fig. 4D) and thus are not reasonably ascribable to current spread to extrathalamic descending fibers.

The MLR region is not only implicated in locomotion through the reticulospinal system [85], [86] but also in modulating pain behavior including escape responses [87]. Our initial idea was that the SRD could directly project to the MLR and thus modulate escape responses through the reticulospinal system but the absence of antidromic activation from the MLR and the long-latency of the orthodromically-induced activity suggest polysynaptic MLR-SRD interactions.

### 3) Effects of LC, PAG, NRM and NRGc stimulation

The data from this series demonstrated antidromic activation of SRD neurons from the LC, the NRM and the NRGc but not from the PAG, corroborating previous results from rodents for the first three structures [10], [14], [17] but in disagreement with the latter report describing SRD projections to the rat's PAG [17] which may either represent a species difference or, alternatively, if there exist SRD-PAG direct connections in the cat constituted by thin fibers, they could have been escaped to antidromic activation with the stimulating currents used in the present study.

The cat's SRD directly projects to the NRM, the LC as well as to the NRGc (this work), structures that participate in felinés nociceptive transmission [18], [64], [88]–[90] and motor behavior [59], [65], [91]–[93]. Accordingly, the SRD may be involved in regulating nociceptive transmission and motor responses initiated by noxious stimuli both directly through SPr fibers, and indirectly through raphespinal, coeruleospinal and reticulospinal fibers.

Finally, the SRD has also the potential capability to integrate multiple behavioral responses associated with the functional organization of the PAG since stimulation at all rostro-caudal and dorso-ventral PAG sites increased the firing activity of SRD cells.

## Conclusion

Taking conjointly, the three series of data from the present study show that the SRD receives information from the thalamus, the LC, the NRM, the NRGc, the MLR and the PAG; entitling SPr neurons to simultaneously spread this information not only throughout the spinal cord but also, via collaterals, to or through the NRGc, the LC and the NRM. The SRD can thus influence spinal cord circuitry both directly and through parallel pathways, probably playing a double functional role aiding to regulate ascending noxious transmission as well as motor behaviors initiated by painful stimuli. This would constitute a supraspinal pain modulatory-and-motor network probably playing an active role in the regulation and performance of nociceptive responses.
